# The electrophysiological index can effectively predict subsequent coronary artery aneurysm in children with Kawasaki disease

**DOI:** 10.20407/fmj.2023-001

**Published:** 2023-08-28

**Authors:** Daijiro Suzuki, Takanori Suzuki, Masayuki Fujino, Yumiko Asai, Arisa Kojima, Hidetoshi Uchida, Kazuyoshi Saito, Hirofumi Kusuki, Yuanying Li, Hiroshi Yatsuya, Tsuneaki Sadanaga, Tadayoshi Hata, Tetsushi Yoshikawa

**Affiliations:** 1 Department of Pediatrics, Fujita Health University, School of Medicine, Toyoake, Aichi, Japan; 2 Clinical Laboratory, JCHO-Chukyo Hospital, Nagoya, Aichi, Japan; 3 Department of Public Health and Health Systems, Nagoya University Graduate School of Medicine, Nagoya, Aichi, Japan; 4 Seigato Hospital, Kumamoto, Kumamoto, Japan; 5 Department of Clinical Laboratory, Fujita Health University Bantane Hospital, Nagoya, Aichi, Japan

**Keywords:** Kawasaki disease, Coronary artery aneurysm, T-peak to T-end interval, QT interval

## Abstract

**Objectives:**

The Gunma score is used to predict the severity of Kawasaki disease (KD), including coronary artery aneurysm (CAA) as a cardiac complication, in Japan. Additionally, the characteristic ratio of ventricular repolarization (T-peak to T-end interval to QT interval [Tp-e/QT]) on a surface electrocardiogram reflects myocardial inflammation. This study aimed to determine whether the Tp-e/QT can be used to predict CAA in children with KD.

**Methods:**

We analyzed chest surface electrocardiograms of 112 children with KD before receiving intravenous immunoglobulin therapy using available software (QTD; Fukuda Denshi, Tokyo, Japan).

**Results:**

The Tp-e/QT (lead V5) was positively correlated with the Gunma score (r=0.352, p<0.001). The Tp-e/QT was larger in patients with CAA (residual CAA at 1 month after onset) than in those without CAA (0.314±0.026 versus 0.253±0.044, p=0.003). A receiver operating characteristic curve analysis was performed to assess whether the Gunma score and Tp-e/QT could predict subsequent CAA. The area under the curve of the Gunma score was 0.719 with the cutoff set at 5 points. The area under the curve of the Tp-e/QT was 0.892 with a cutoff value of 0.299. The fit of the prediction models to the observed probability was tested by the Hosmer–Lemeshow test with calibration plots using Locally weighted scatterplot smoothing (LOESS) fit. The Gunma score (p=0.95) and Tp-e/QT (p=0.95) showed a good fit.

**Conclusions:**

The Tp-e/QT is a useful biomarker in predicting coronary aneurysm complications in KD.

## Introduction

Kawasaki disease (KD) is a cryptogenic acute febrile illness that causes systemic vasculitis, myocardial inflammation, and coronary vasodilation. In advanced countries, KD is the most common cause of acquired heart disease in children. A recent nationwide survey in Japan showed that 2.2% of patients with KD developed coronary artery aneurysm (CAA) as a cardiac complication (≥1 month after onset).^1^ In children who develop CAA, the dispersion of ventricular repolarization due to ischemic myocardial damage is thought to increase.^2,3^ A previous study indicated that an increase in the dispersion of the late-stage repolarization (T-peak to T-end [Tp-e]) interval might be used to predict the occurrence of fatal arrhythmia in patients with Brugada syndrome.^4^ Another study reported that the ratio of the Tp-e interval to the QT interval (Tp-e/QT) was increased in pediatric patients with hypertrophic cardiomyopathy who developed fatal arrhythmias.^5^ These reports indicate that an increase in the Tp-e/QT, which represents the dispersion of ventricular repolarization, promotes the functional reentry of cardiac muscles contributing to the onset of these arrhythmias.^6^ We previously reported that the Tp-e/QT obtained from an electrocardiogram (ECG) in the acute phase of KD was positively correlated with inflammation.^7^ We further reported that the QT variability index, which is another marker of myocardial repolarization abnormalities, was high in the fever stage of KD.^8^ These findings suggest the existence of abnormal myocardial repolarization in patients with KD because it is a systemic inflammatory disease involving the heart.

The Gunma scoring system is a risk-scoring system that is frequently used to predict the severity of KD and CAA.^9^ Each component of this scoring system and its cutoff values are defined as follows: serum sodium concentrations, ≤133 mmol/L; time elapsed after onset at diagnosis, ≤4 days; aspartate aminotransferase (AST) concentrations, ≥100 U/L; neutrophil ratio among all white blood cells, ≥80%; platelet count, ≤30×10^4^/μL; C-reactive protein (CRP) concentrations, ≥10 mg/dL; and 1 point each for patients aged ≤12 months. In Japan, a Gunma score of ≥5 is used to predict the severity of KD, and active acute-phase treatment is being promoted to improve the therapeutic results.

The present study aimed to determine whether the Tp-e/QT can be used as a biomarker to predict the severity of KD and CAA as a cardiac complication in pediatric patients.

## Methods

This study included 126 patients with KD whose ECGs were recorded before starting treatment at Fujita Health University. A standard 12-lead ECG taken at rest was obtained from admission within 24 hours before the first intravenous immunoglobulin (IVIG) treatment (4–10 days of illness; median, 5.2 days). Patients whose steady ECG recording was not available because of intense movement or crying and patients with a high heart rate (≥180 beats/minute) due to overlapping of the T-end and P waves were excluded from the study. The patients’ medical records were retrospectively analyzed. The treatment of all patients conformed to the guidelines of the American Heart Association.^10,11^ In patients with a Gunma score of ≥5 (patients with predicted IVIG resistance), a therapeutic regimen using IVIG (2 g/kg) along with prednisolone was implemented in principle. In children with a Gunma score of <5, only IVIG (2 g/kg) was administered (prednisolone was not used in principle). Additionally, aspirin was administered at a dosage of 50 mg/kg/day for the first 5 to 10 days, and the dose was reduced in the following 4 to 6 weeks (5 mg/kg). Children who did not achieve defervescence within 24 hours after the initial IVIG administration were categorized as patients with initial therapy resistance. An additional IVIG regimen was administered to children who had a persistent fever lasting >48 hours after the initial IVIG administration or patients who developed a recurrent fever associated with KD symptoms after a certain apyretic interval. No catecholamines or diuretics were administered to treat cardiac failure, and no drugs that might affect the QT interval were used. This study was approved by the ethics committee of our institution for epidemiological and clinical research purposes (approval no. HM20-305). Written

informed consent was obtained from the subjects and their parents or guardians.

### Parameters

Laboratory markers, such as the white blood cell count, hematocrit, platelet count, AST concentrations, sodium concentrations, and CRP concentrations (required to calculate the Gunma scores of patients with acute-phase KD) were measured before the initial treatment.

**Figure 1:**
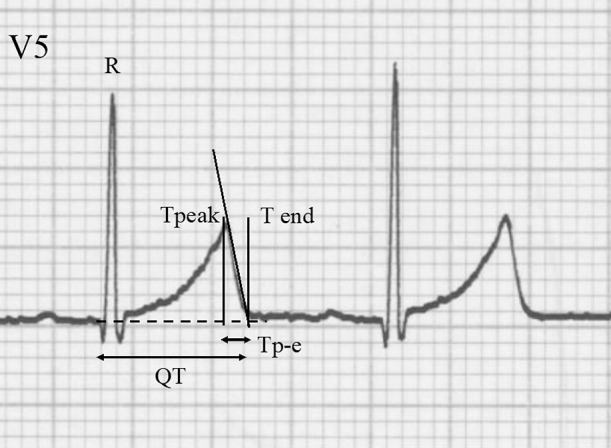
Measurement of QT and Tp-e intervals The end of the T wave was measured from the intersection of the tangent to the repolarization slope with the isoelectric line (dashed line). Tp-e, T-peak to T-end.

The patients’ 12-lead ECGs were recorded by activating hum, high-cut, and drift filters stored on a computer disk by an independent investigator who was unaware of the patients’ clinical profiles. In each patient, the RR interval, start of the Q wave, and peak of the T wave were measured using analytical software (QTD; Fukuda Denshi Co., Ltd., Tokyo, Japan). The end of the T wave was determined by the tangent line method (Figure 1). The Bazett formula and Fridericia formula were used to calculate the corrected QT interval (QTcB and QTcF, respectively). The Tp-e/QT at the chest leads was then calculated as the characteristic ratio of ventricular repolarization.^6^ The means of three consecutive heartbeats were used for the analyses for all ECG indices. The correlations between the Tp-e/QT, Gunma score, and other relevant parameters were subsequently investigated. There are two reasons for adopting lead V5 for Tp-e/QT analysis in this study. First, the guidelines of the American Heart Association, American College of Cardiology Foundation, and Heart Rhythm Society recommend using lead V5 or V6 for the measurement of QT interval.^12^ Second, in infants, there is empirical recognition that the voltage in a V6 lead is low owing to the positional relationship between the thoracic cage and the heart. Therefore, a V5 lead is a reasonable choice for the analysis.

### Coronary artery abnormalities

All patients underwent echocardiography at least once weekly, on admission, during hospitalization, and in the outpatient clinic at week 4 during the follow-up observation period. In accordance with the American Heart Association guidelines, CAAs were defined as coronary vasodilation lesions with a Z-score (the standard deviation unit from the mean inner diameter normalized to the surface area) of ≥2.5.^5^ Transthoracic ultrasonography was performed before treatment to detect coronary lesions, and if at least one of the four measurement sites (coronary classification segments 1, 5, 6, and 11) recorded a Z-score of >2.5, the patient was diagnosed with CAA. Moreover, patients who had CAA only in the acute phase were categorized into the transient CAA (TCAA) group, and patients who had residual CAA 1 month after the onset were categorized into the CAA group.

### Statistical analysis

Data are presented as the mean±standard deviation, median (interquartile range), or percentage, as appropriate. Comparisons between two groups of data were made with the unpaired Student t test or the Mann–Whitney U test, as appropriate. Comparisons among three groups of data were made with the Steel–Dwass test. A Pearson correlation analysis was also performed. A receiver operating characteristic (ROC) analysis was performed to assess whether the Gunma score and Tp-e/QT could be used to predict subsequent CAA as a cardiac complication of KD. DeLong’s test was performed to compare the areas under the curve (AUCs) of both ROC curves. The goodness of fit of the prediction models to the observed probability was tested by the Hosmer–Lemeshow test. Subjects were grouped according to six quantiles of the predicted probability in this analysis. Calibration plots using LOESS fit were also used to graphically evaluate the degree of agreement between the predicted and observed probability of CAA. SAS 9.4 (SAS Institute Inc., Cary, NC, USA) was used for these analyses. All other statistical analyses were performed using JMP Statistical Analysis Software, version 14. SW (SAS Institute Inc., Cary, NC, USA). In all analyses, the significance level was set at p<0.05.

## Results

### Patients’ characteristics and clinical outcomes

**Figure 2:**
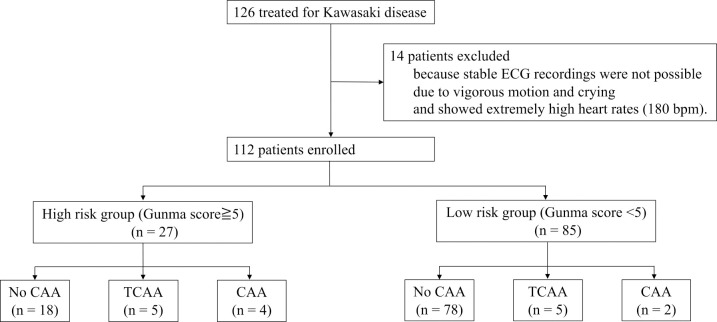
Participants included in this study Initially, 126 patients with Kawasaki disease were included. Fourteen patients were excluded because their ECGs did not show analyzable waveforms. Finally, 112 patients were included in this study. There were 27 patients in the high-risk group (Gunma score of ≥5), among whom TCCA was observed in 5 patients and CAA in 4 patients. There were 85 patients in the low-risk group (Gunma score of <5), among whom TCAA was observed in 5 patients and CAA in 2 patients. ECG, electrocardiogram; TCAA, transient coronary artery aneurysm; CAA, coronary artery aneurysm.

Figure 2 shows a flow chart of patients’ inclusion, exclusion, and clinical outcomes in this study. Initially, 126 patients with KD who were hospitalized at our institution were included. Fourteen patients were excluded because an analyzable ECG could not be obtained; consequently, 112 patients were enrolled. Twenty-seven patients had a Gunma score of ≥5 points, and 13 (48.1%) of these patients were administered IVIG and prednisolone. Eighty-five patients had a Gunma score of <5 points, and nine (10.6%) of these patients were administered IVIG and prednisolone. Among patients with IVIG resistance, five (18.5%) patients had a Gunma score of ≥5 points, and seven (8.2%) patients had a Gunma score of <5 points. TCCA was observed in five (18.5%) patients in the high-risk group and in five (5.9%) patients in the low-risk group. CAA was observed in four (14.8%) patients in the high-risk group and in two (2.4%) patients in the low-risk group.

Table 1 shows the patients’ characteristics. The patients comprised 68 boys and 44 girls, and their mean age was 1.94±1.62 years. No significant difference in age or sex was observed between patients with a Gunma score of ≥5 and those with <5. However, TCAA and CAA were frequently observed in patients with a Gunma score of ≥5 (p=0.045 in TCAA, p=0.012 in CAA versus a Gunma score <5).

### Relationships between the Tp-e/QT (V1–V6) and each indicator

Table 2 shows the relationships between the Tp-e/QT and several parameters in individual chest leads. Regarding Tp-e/QT (lead V5), sodium concentration was negatively correlated (r=–0.405, p<0.001), whereas CRP concentration and Gunma score were positively correlated (r=0.292, p=0.002, r=0.352, p<0.001, respectively).

### Comparison of ECG parameters and laboratory data in patients with low versus high Gunma scores and in patients with TCAA and CAA versus no CAA

Table 3 shows the comparison of ECG parameters and laboratory data in patients with low versus high Gunma scores and in patients with TCAA and CAA versus no CAA. In patients with a high Gunma score, the AST and CRP concentrations were significantly higher (p<0.001 and p<0.001) and the sodium concentration and platelet count were significantly lower (p<0.001 and p<0.001) than those in patients with a low Gunma score. Notably, the Tp-e/QT was positively correlated with the Gunma score (r=0.352, p<0.001) and was larger in patients with a score of ≥5 than <5 (0.277±0.036 vs. 0.254±0.046, p=0.041). Tp-e was longer, and CRP was higher in patients with than without CAA (p=0.008 and p=0.015, respectively). patients with CAA tended to show a longer QTc than patients without CAA (QTcB: no CAA, 397.2±28.1; CAA, 419.6±41.1; p=0.326; and QTcF: no CAA, 342.6±30.1; CAA, 372.1±53.1; p=0.341).

### Comparison of the Gunma score and Tp-e/QT in patients with TCAA and CAA versus no CAA

Table 4 shows the comparison of the Gunma score and Tp-e/QT in patients with TCAA and CAA versus patients with no CAA. The mean Gunma score was significantly higher in patients with TCAA and CAA than in patients with no CAA (both p<0.05). The Tp-e/QT was significantly higher in patients with TCAA and CAA than in patients with no CAA (both p<0.05).

### ROC analyses of the Gunma score and Tp-e/QT for predicting TCAA and CAA

**Figure 3:**
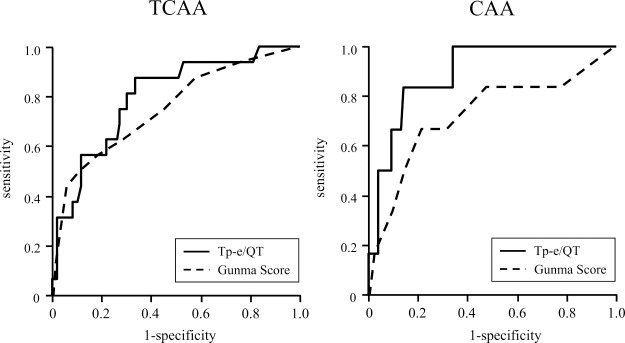
Comparison of the AUC using receiver operating characteristic analysis In the TCAA group (left panel), the AUC for the Tp-e/QT was comparable to that for the Gunma score. In the CAA group (right panel), the AUC for the Tp-e/QT was numerically larger than that for the Gunma score, but it did not reach statistical significance (p=0.058). See Table 5 for details. AUC, area under the curve; TCAA, transient coronary artery aneurysm; Tp-e, T-peak to T-end; CAA, coronary artery aneurysm.

Figure 3 and Table 5 show the results of the ROC analyses of the Tp-e/QT and Gunma score for the prediction of CAA. The AUCs of the Gunma score and Tp-e/QT for the prediction of TCAA were 0.746 and 0.798, respectively, without statistical significance. The AUC of the Tp-e/QT for the prediction of CAA was 0.892, which was numerically higher than that of the Gunma score (0.719), but it did not reach significance (Delong’s test, p=0.058).

### Goodness of fit of the prediction models for CAA using the Gunma score and Tp-e/QT

**Figure 4:**
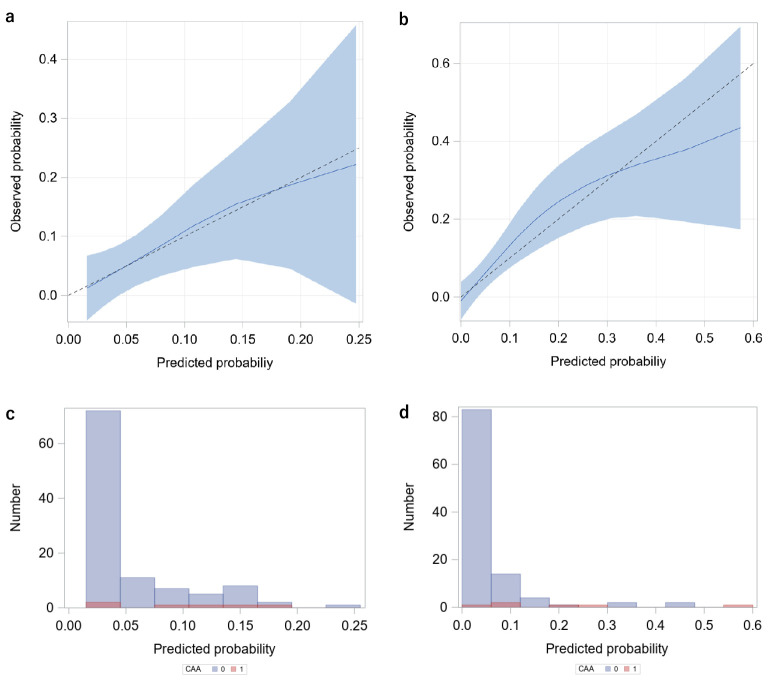
Calibration plots of the Gunma score and Tp-e/QT in predicting CAA LOESS calibration plots are shown in the upper panels (left [a]: Gunma score, right [b]: Tp-e/QT). The dotted straight line, solid line curve, and shaded zone indicate the diagonal line representing the observed probability=predicted one, LOESS calibration curve, and 95% confidence interval of the LOESS fit, respectively. Histograms of the predicted probability for the presence of CAA in patients with CAA (CAA [1], red) and those without CAA (CAA [0], blue) are shown in the lower panels (left [c]: Gunma score, right [d]: Tp-e/QT]. Tp-e, T-peak to T-end; CAA, coronary artery aneurysm; LOESS, Locally weighted scatter-plot smoothing.

Predicted probabilities for the presence of CAA by the Gunma Score (Figure 4a) and by the Tp-e/QT (Figure 4b) generally matched the observed probabilities. This occurred despite the wide confidence intervals in the higher probability ranges as indicated by the small number of individuals, especially in the higher probability ranges (Figure 4c and 4d). The Hosmer–Lemeshow test also indicated the goodness of fit (p=0.73 for the Gunma score and p=0.95 for the Tp-e/QT).

## Discussion

This study evaluated the relationships between the Tp-e/QT and the severity of KD using the Gunma score. The Tp-e/QT was positively correlated with the Gunma score, and the Tp-e/QT was larger in patients with a score of ≥5 than in those with a score of <5. In patients with CAA (including TCAA), the Tp-e/QT was significantly higher than that in patients with no CAA. Although the number of patients who developed CAA was small, the ROC analysis showed that the predictive efficacy of the Tp-e/QT for TCAA was comparable to or higher than that of the Gunma score (Figure 3, Table 5). We also confirmed the goodness of fit of the prediction models by the Hosmer–Lemeshow test and the calibration plot (Figure 4).

The Tp-e/QT index is defined as the ratio between the repolarization heterogeneity of late-phase ventricular repolarization (Tp-e interval) and myocardial repolarization (QT interval).^6^ Previous studies of the dispersion of ventricular repolarization demonstrated that the Tp-e/QT was effective in evaluating the propensity for arrhythmia, including in patients with myocardial infarction, hypertrophic cardiomyopathy, and Brugada syndrome.^13–15^ We previously found that the Tp-e/QT obtained from ECG records in patients with acute-phase KD was positively correlated with fever (body temperature) and CRP concentrations in the simultaneous phase.^7^ We also reported that the QT variability index was high in the fever stage.^8^

The QT interval, which represents myocardial repolarization, is modulated in various systemic inflammatory diseases. Previous studies have shown that the QT interval is increased in patients with systemic inflammatory disease who develop acquired long QT syndrome, and the degree of the increase is dependent on the levels of inflammatory cytokines.^16,17^

The Tp-e interval is considered to reflect the physiological difference in the action potential duration between the endocardial and epicardial myocardium.^18^ In the normal myocardium, there is heterogeneity of ion channels in the endocardium and epicardium. In this situation, inflammatory substances generated in patients with KD, such as cytokines, can further modulate ion channels, thereby increasing the repolarization heterogeneity. In the present study, patients with CAA tended to show a longer QTc than patients without CAA. This result contradicts a recent report stating that pediatric patients with KD who developed CAA displayed a short QTc.^19^ However, many other reports support our findings. Li and Rozanski^20^ showed that cytokines affected calcium handling and prolonged the duration of the action potential. Similarly, a clinical study conducted by Lazzerini et al.^21^ showed that cytokines mediated myocardial electrical remodeling, and considerable QTc prolongation occurred in patients with systemic inflammation. Moreover, recent experiments using the *Lactobacillus casei* cell wall extract-induced KD vasculitis model suggested that interleukin 1 was increased in sympathetic neurons, myocardial cells, and ganglions, possibly by increasing myocardial electrical instability.^22^ The results of the above-mentioned studies also suggest that inflammation prolongs the myocardial repolarization duration and increases the repolarization heterogeneity in patients with KD.

In Japan, several scoring systems for evaluating the severity of KD have been reported.^23–25^ The Gunma scoring system is most widely used for stratifying patients when determining the indication for strong initial treatment. Moreover, this scoring system is accepted as the gold standard for predicting CAA as a complication of KD.^26^ The ROC analysis in this study indicated the effectiveness of the Gunma score. However, the Tp-e/QT showed a trend toward a better AUC value than that of the Gunma score. Because of the simplicity and noninvasiveness of the Tp-e/QT, it could be a promising biomarker for the prediction of CAA in children with KD.

### Limitations

This study has two main limitations. First, we were unable to analyze the QT intervals from the ECG records when the heart rate was high (≥180 beats/minutes) because the end of the T wave overlapped with the ascending limb of the P wave. Second, this was a retrospective study including a limited number of patients from a single center. Therefore, our study might contain several potential biases. Specifically, because the number of patients with TCAA and CAA was small, the predictive efficacy of the Tp-e/QT might not have been fully evaluated. Therefore, prospective, multicenter studies are required.

## Conclusion

The Tp-e/QT is correlated with the severity of KD as evaluated by the Gunma score. This study suggests that the Tp-e/QT represents the modulation of myocardial repolarization in cardiac inflammation. Furthermore, the Tp-e/QT could be a useful biomarker for predicting CAA as a complication of KD.

## Figures and Tables

**Table1:** Patients’ characteristics

	number of patients	Age (average±SD)	Sex (M:F)	Gunma score	TCAA	CAA
All subjects	112	1.94±1.62	68:44	2.8±2.4	10 (8.9%) M:F 8:3	6 (5.4%) M:F 4:1
Gunma score <5	85	1.85±1.67	52:33	1.7±1.4	5 (5.9%) M:F 4:1	2 (2.4%) M:F 1:1
Gunma score ≥5	27	2.00±1.53	16:11	6.4±1.1	5 (18.5%) M:F 4:2	4 (14.8%) M:F 3:0

Values are expressed as n, n (%), or the mean±standard deviation. Comparisons between two groups of data were made with the chi-square test or unpaired Student t-test. No significant difference in age or sex was observed between patients with a Gunma score of ≥5 and <5. M, male; F, female; TCAA, transient coronary artery aneurysm; CAA, coronary artery aneurysm.

**Table2:** Relationships between the Tp-e/QT (V1–V6) and some variables

	AST	Sodium	CRP	Plt	BT (°C)	Gunma score
V1 (Tp-e/QT)	r: 0.405	–0.131	0.177	–0.044	0.231	0.173
	p: <0.001*	0.171	0.065	0.652	0.015*	0.072

V2	–0.091	–0.017	0.069	0.077	0.035	0.070
	0.343	0.862	0.476	0.422	0.714	0.470

V3	0.058	0.085	0.021	–0.062	0.071	0.012
	0.545	0.378	0.827	0.522	0.460	0.900

V4	0.235	–0.303	0.236	–0.015	0.081	0.282
	0.014*	0.001*	0.013	0.876	0.402	0.003*

V5	0.185	–0.405	0.292	–0.033	0.173	0.352
	0.053	<0.001*	0.002*	0.734	0.071	<0.001*

V6	0.236	–0.330	0.209	0.006	0.161	0.359
	0.013*	<0.001*	0.028*	0.951	0.093	<0.001*

AST, aspartate transaminase; CRP, C-reactive protein; Plt, platelet count; BT, body temperature.

**Table3:** Comparison of electrocardiographic parameters and laboratory data between patients with a Gunma score of <5 versus ≥5 (left) and between patients with TCAA and CAA versus no CAA

	Gunma score <5	Gunma score ≥5	No CAA	TCAA	CAA
number (n)	85	27	96	10	6
HR (beat/min)	149.1±31.3	148.5±24.7	149.6±28.8	153.5±34.6	131.0±35.9
RR (ms)	421.8±97.6	417.5±86.6	417.5±90.1	410.8±99.8	490.2±143.9
QT (ms)	257.8±41.3	260.5±39.5	255.8±37.0	261.4±47.8	294.4±69.9
QTcB (ms)	398.0±29.8	403.8±29.9	397.2±28.1	408.4±35.8	419.6±41.1
QTcF (ms)	343.9±33.2	348.5±32.4	342.6±30.1	351.5±40.0	372.1±53.1
Tp-e (ms)	61.7±14.8	66.0±13.5	61.0±13.0	67.2±17.7	83.2±17.8^#^
Tp-e/QT	0.254±0.046	0.277±0.036*	0.253±0.044	0.287±0.033^#^	0.314±0.026^#^
AST (U/L)	53.0±99.4	183.1±174.4*	68.9±110.2	181.3±197.1	171.8±245.7
Sodium (mEq/L)	135.7±2.6	131.7±2.4*	135.1±2.9	132.5±3.4	133.2±2.8
CRP (mg/dL)	6.9±4.5	10.1±3.9*	7.4±4.3	6.9±4.5	13.6±4.7^#^
Platelet (10^4^/μL)	36.9±11.1	30.1±7.8*	35.5±11.0	33.6±8.6	33.2±11.3
BT (℃)	38.6±0.9	38.9±0.9	38.6±1.0	39.0±0.4	38.4±0.6

Values are expressed as the mean±standard deviation. HR, heart rate; RR, RR interval; Tp-e, T-peak to T-end; AST, aspartate transaminase; CRP, C-reactive protein; BT, body temperature; CAA, coronary artery aneurysm; TCAA, transient coronary artery aneurysm. * p<0.05 versus a Gunma score of <5; ^#^ p<0.05 versus no CAA.

**Table4:** Comparison between the Gunma score and Tp-e/QT in patients with TCAA and CAA versus no CAA

(n)	No CAA (96)	TCAA (10)	CAA (6)
Gunma score	2.5±2.2	4.8±2.7*	4.8±2.9
Tp-e/QT	0.253±0.044	0.287±0.033*	0.314±0.028*

Values are expressed as the mean±standard deviation. TCAA, transient coronary artery aneurysm; CAA, coronary artery aneurysm. * p<0.05 versus no CAA.

**Table5:** Results of receiver operating characteristic analysis of the Gunma score and Tp-e/QT for predicting TCAA and CAA

TCAA (10)	Gunma Score	Tp-e/QT	
AUC [95%CI]	0.746 [0.606–0.887]	0.798 [0.680–0.915]	p=0.601
Cutoff	5	0.274	
Sensitivity	56.3%	87.5%	
Specificity	81.3%	66.7%	

CAA (6)	Gunma Score	Tp-e/QT	

AUC [95%CI]	0.719 [0.454–0.984]	0.892 [0.787–0.998]	p=0.058
Cutoff	5	0.299	
Sensitivity	66.7%	83.3%	
Specificity	78.3%	85.9%	

TCAA, transient coronary artery aneurysm; CAA, coronary artery aneurysm; AUC, area under the curve; CI, confidence interval.
